# Supplementary material for: Antibacterial Activity of Bis(4-aminopyridinium) Compounds for Their Potential Use as Disinfectants
Source: Molecules. 2025 Oct 2;30(19):3962. doi: 10.3390/molecules30193962 (PMC12525534; doi:10.3390/molecules30193962)
Supplement: Supplementary file 1 [file molecules-30-03962-s001.zip › molecules-3776459-supplementary.pdf]

Antibacterial activity of bis(4-aminopyridinium) compounds for their potential use as disinfectants

Carolina Arriaza-Echanes [1], Claudio A. Terraza [2], Mateus Frazao[3], Sebastián Reyes-Cerpa[3,4], Loreto Sanhueza [5]* and Pablo A Ortiz [1,6]*

[1] Centro de Nanotecnología Aplicada, Facultad de Ciencias, Ingeniería y Tecnología, Universidad Mayor, Camino La Pirámide 5750, Huechuraba, 8580745, Santiago, RM, Chile; carolina.arriaza@mayor.cl

[2] Research Laboratory for Organic Polymers (RLOP), Department of Organic Chemistry, Pontificia Universidad Católica de Chile, 7820436, Santiago, Chile; cterraza@uc.cl

[3] Centro de Genómica y Bioinformática, Facultad de Ciencias, Ingeniería y Tecnología, Universidad Mayor, Santiago 8580745, Chile; mateus.frazao@mayor.cl

[4] Escuela de Biotecnología, Facultad de Ciencias, Ingeniería y Tecnología, Universidad Mayor, Santiago 8580745, Chile; sebastian.reyes@umayor.cl

[5] Núcleo de Química y Bioquímica, Facultad de Ciencias, Ingeniería y Tecnología, Universidad Mayor, Camino La Pirámide 5750, Huechuraba, 8580745, Santiago, RM, Chile; loreto.sanhueza@umayor.cl

[6] Escuela de Ingeniería en Medio Ambiente y Sustentabilidad, Facultad de Ciencias, Ingeniería y Tecnología, Universidad Mayor, Camino La Pirámide 5750, Huechuraba, 8580745, Santiago, RM, Chile; pablo.ortiz@umayor.cl

Spectroscopic data

1,1’-(Ethane-1,2-diyl)bis(4-aminopyridin-1-ium) bromide (C2)

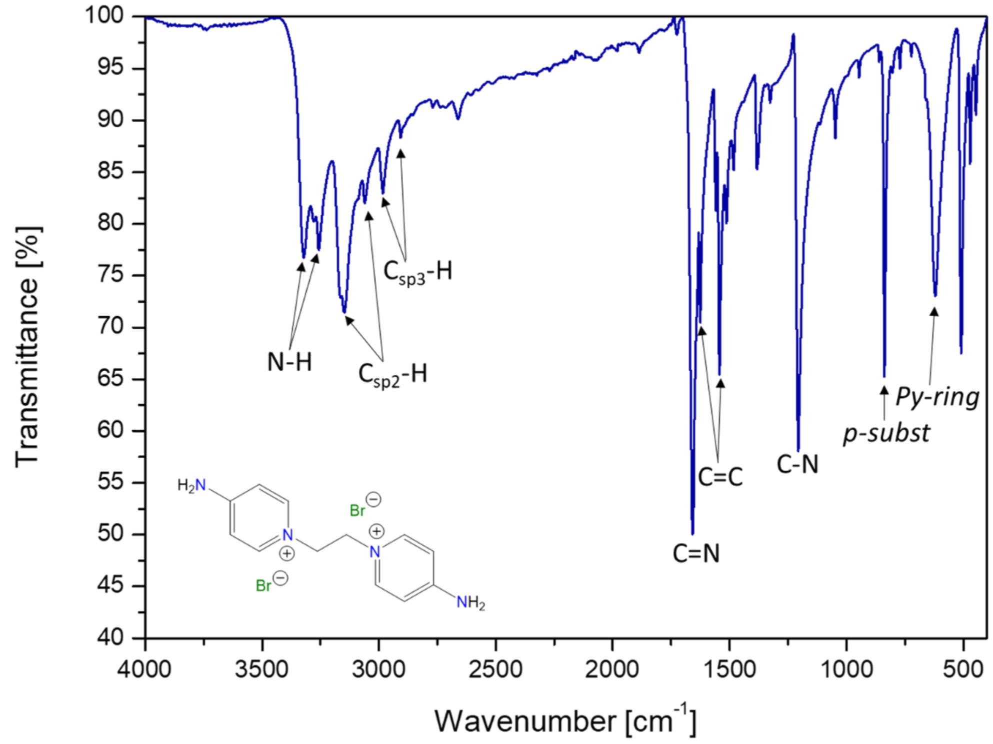

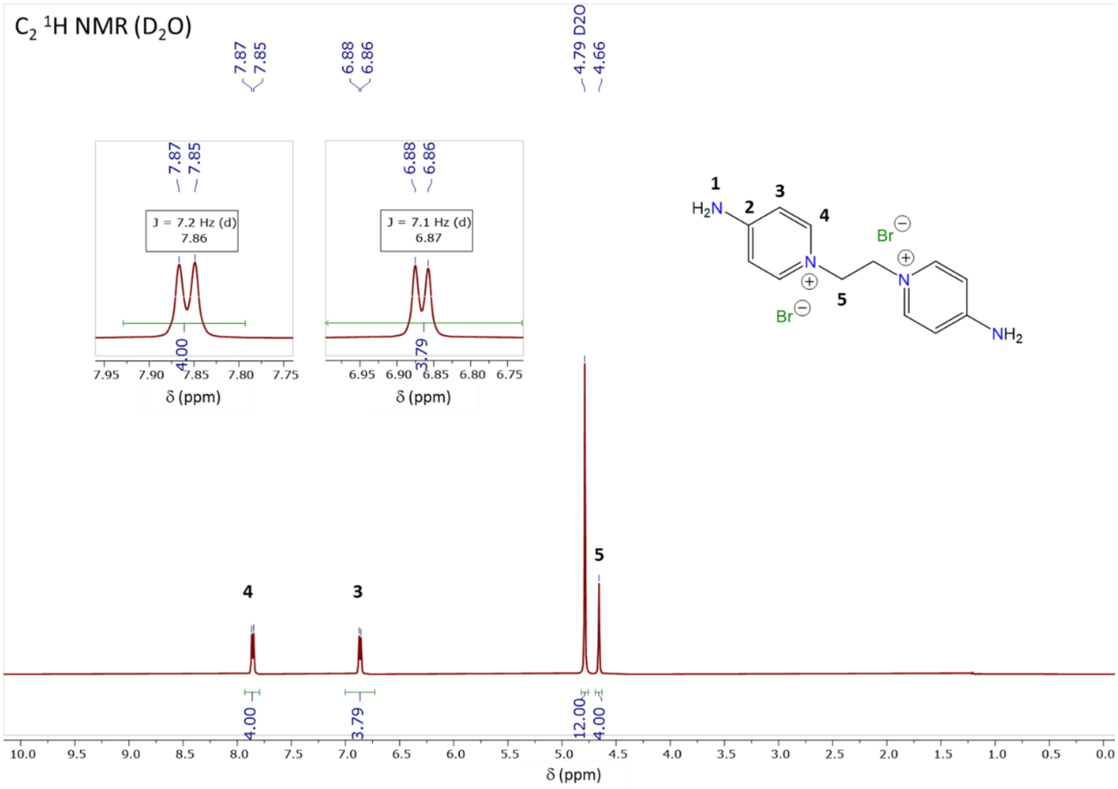

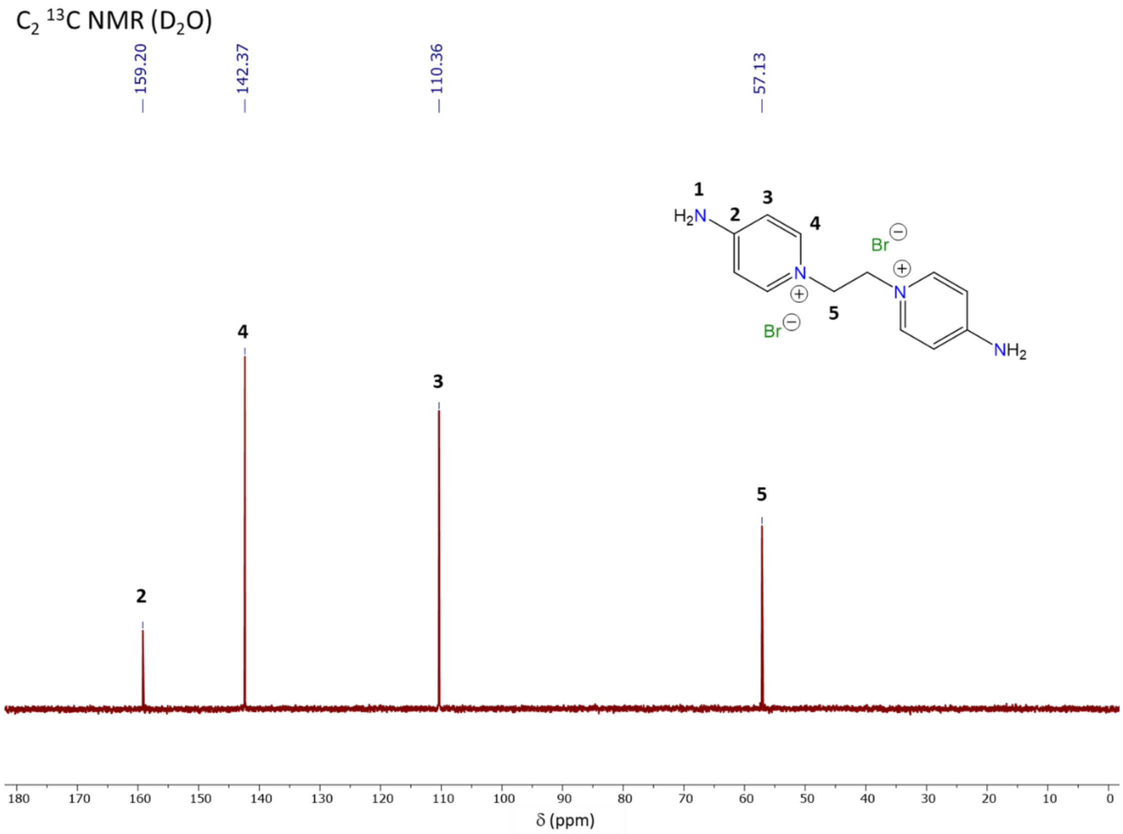

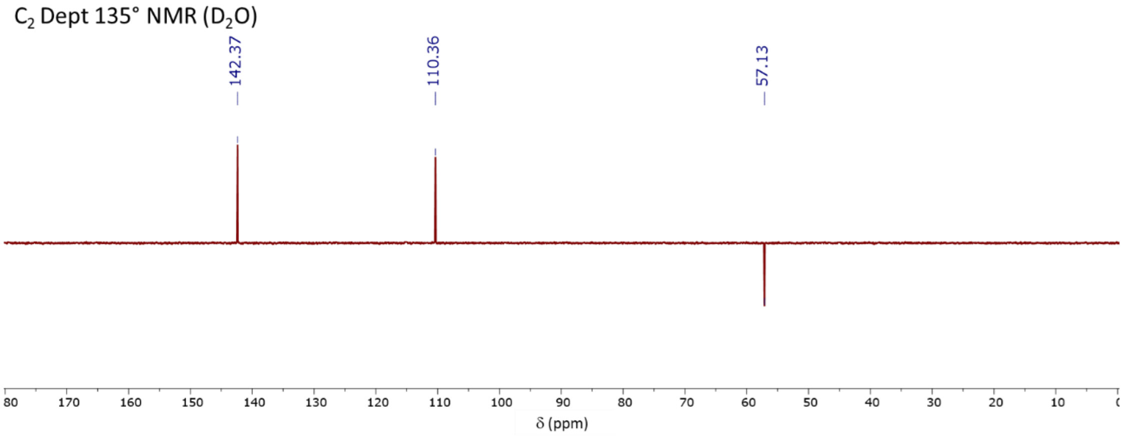

Figure S1. Spectroscopy characterization of 1,1’-(ethane-1,2-diyl)bis(4-aminopyridin-1-ium) bromide (FT-IR, ^{1}H, ^{13}C and Dept 135° NMR spectra).

1,1'-(Propane-1,3-diyl)bis(4-aminopyridin-1-ium) bromide $[C_3(4\text{-}NH_2Py)_2][Br]_2$ (C_3)

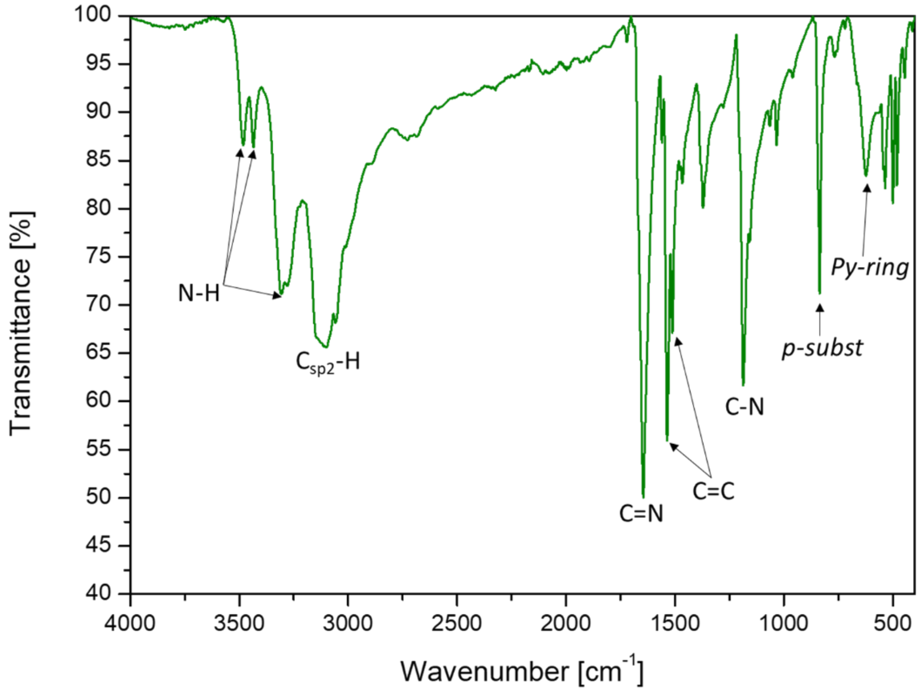

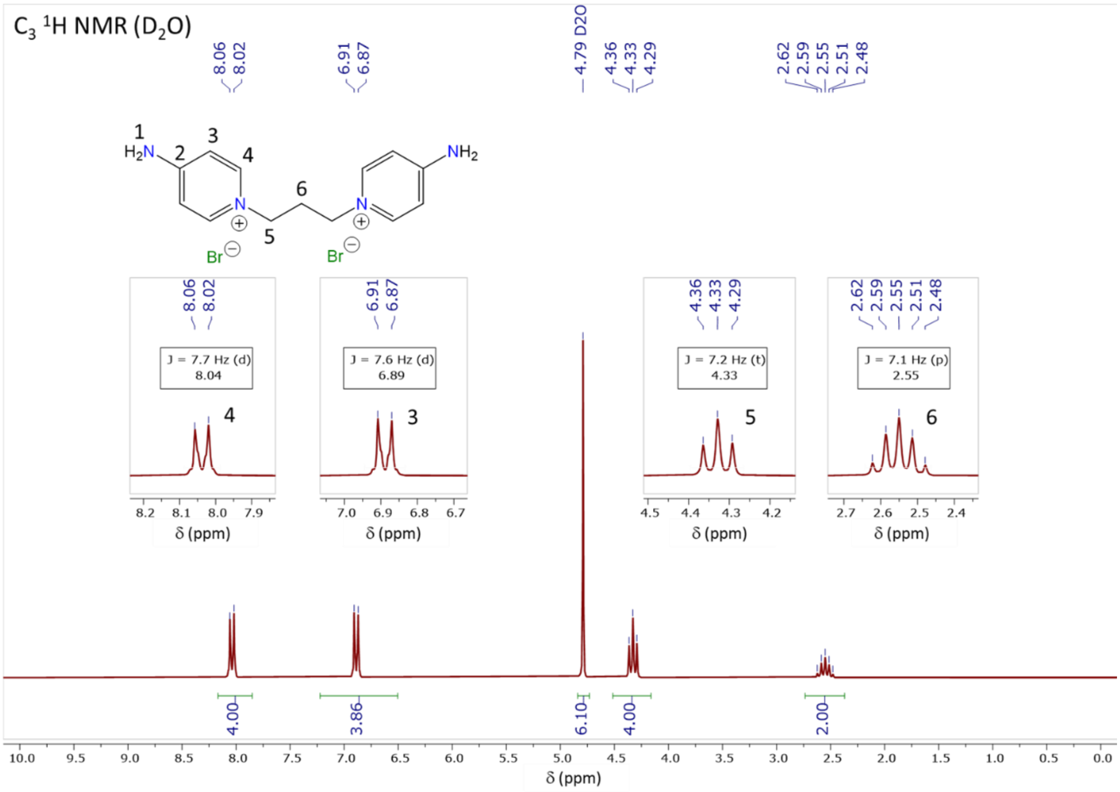

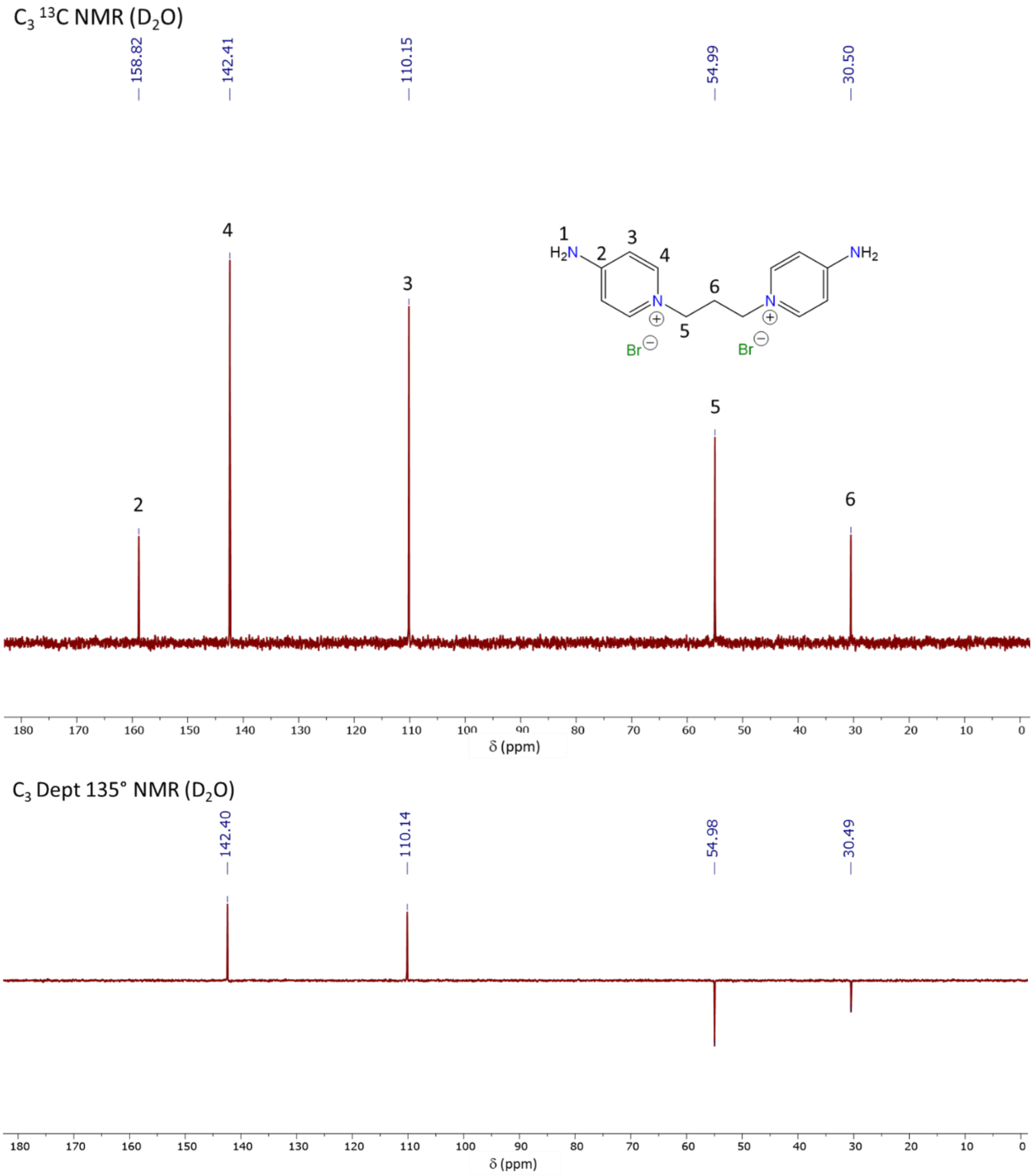

Figure S2. Spectroscopy characterization of 1,1'-(propane-1,3-diyl)bis(4-aminopyridin-1-ium) bromide (FT-IR, ^{1}H, ^{13}C and Dept 135° NMR spectra).

1,1'-(Butane-1,4-diyl)bis(4-aminopyridin-1-ium) bromide [C_4(4-NH_2Py)$_2$][Br]$_2$ (C_4)

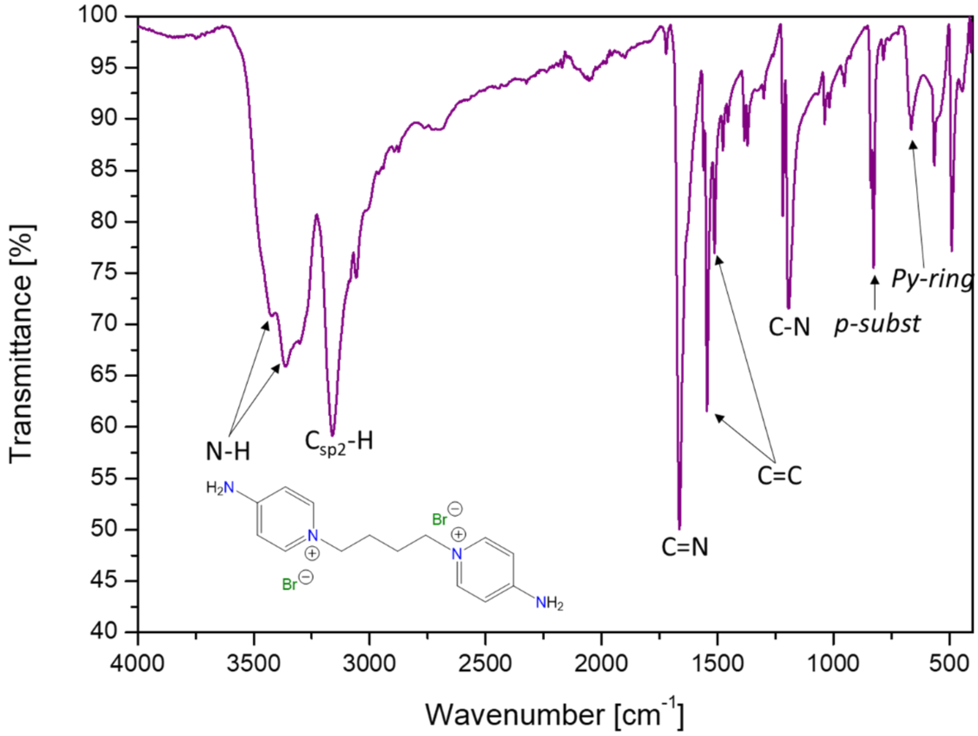

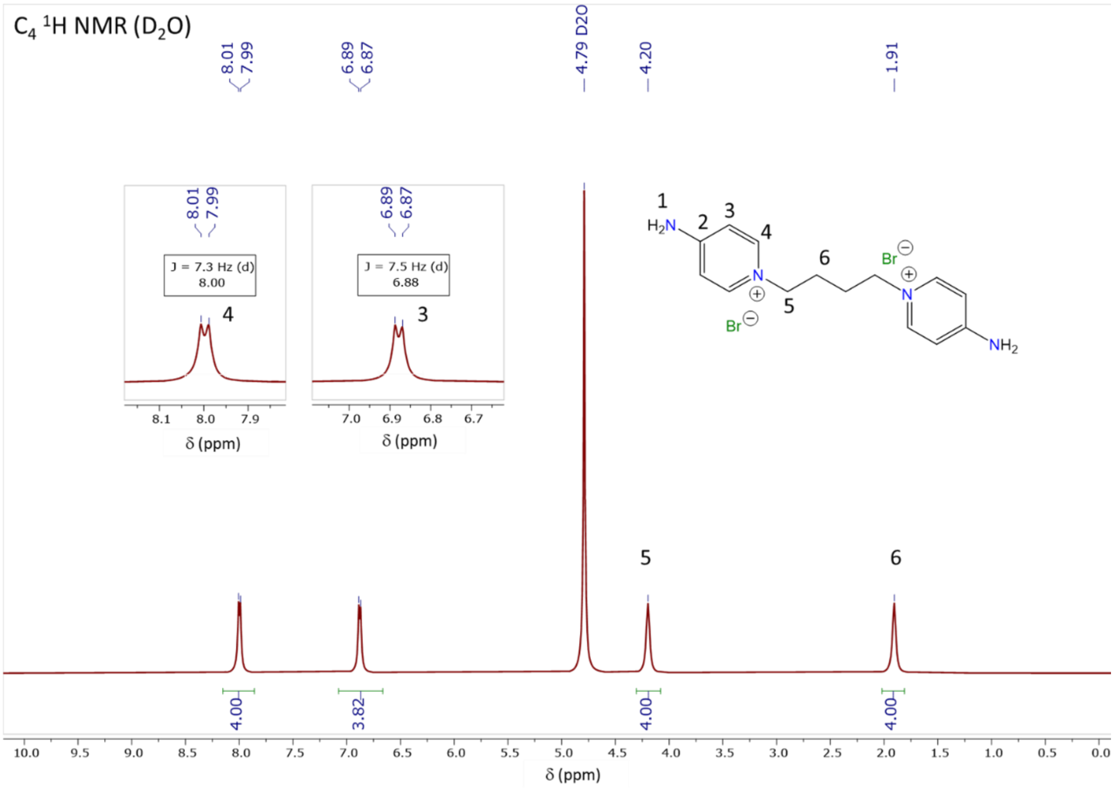

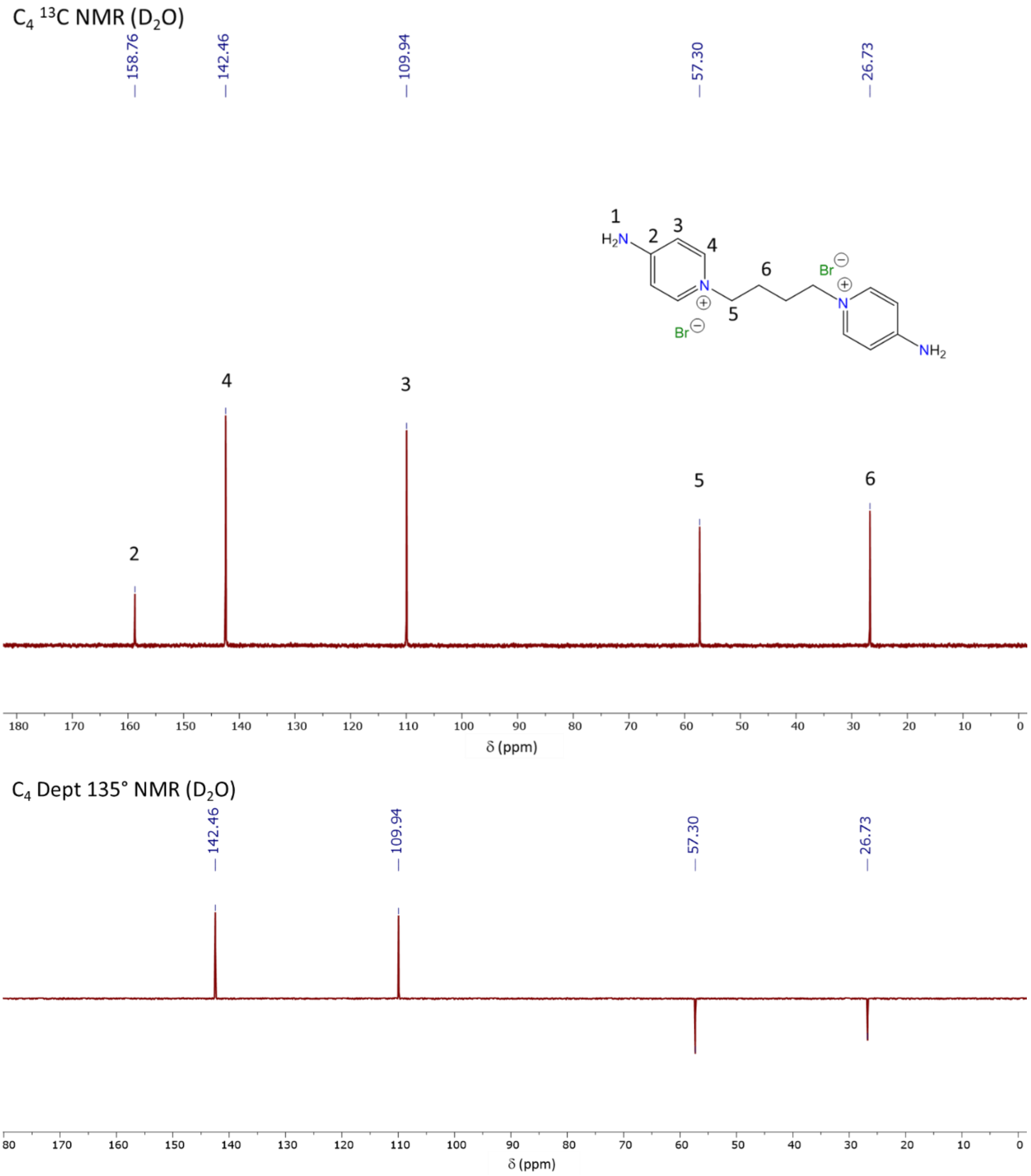

Figure S3. Spectroscopy characterization of 1,1'-(butane-1,4-diyl)bis(4-aminopyridin-1-ium) bromide (FT-IR, ^{1}H, ^{13}C and Dept NMR 135° spectra).

1,1’-(Pentane-1,5-diyl)bis(4-aminopyridin-1-ium) bromide [$C_5(4\text{-}NH_2Py)_2$][Br]$_2$ (C_5)

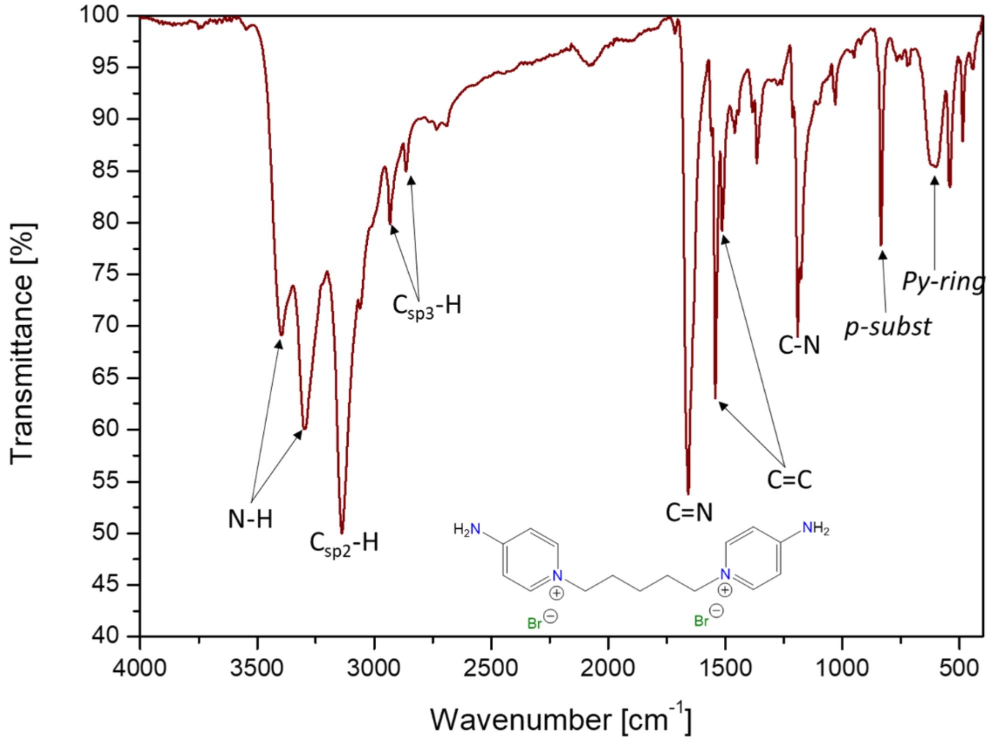

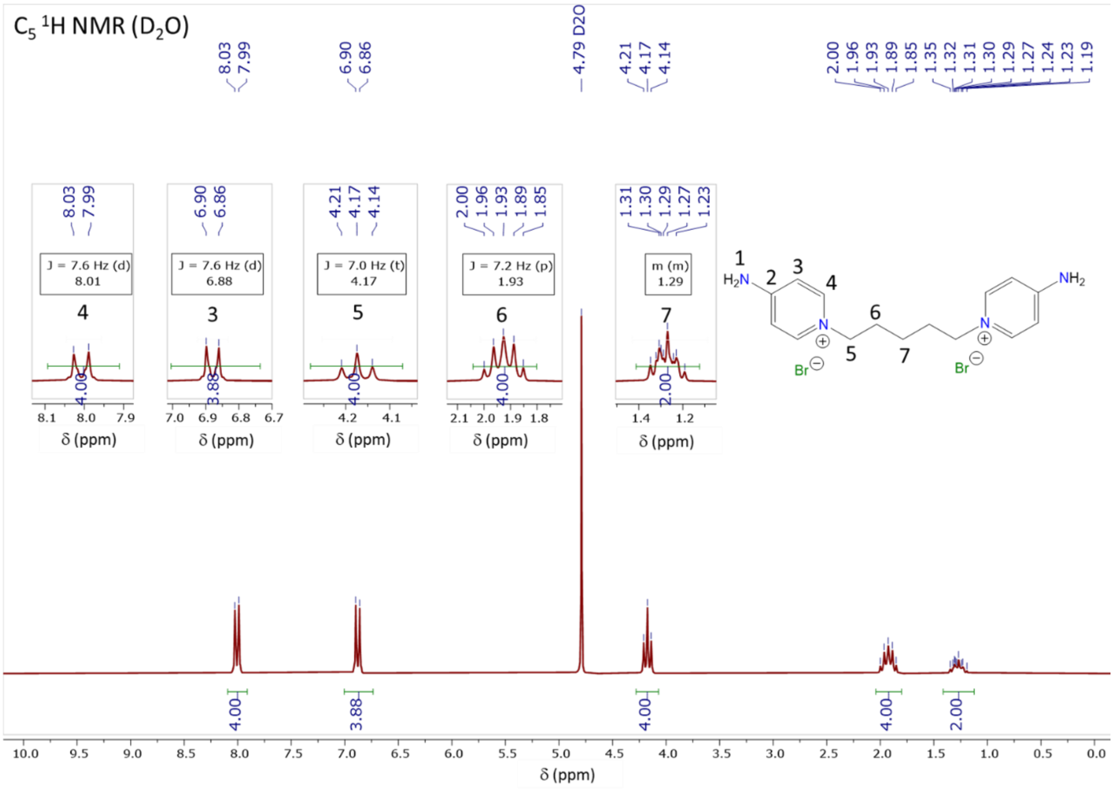

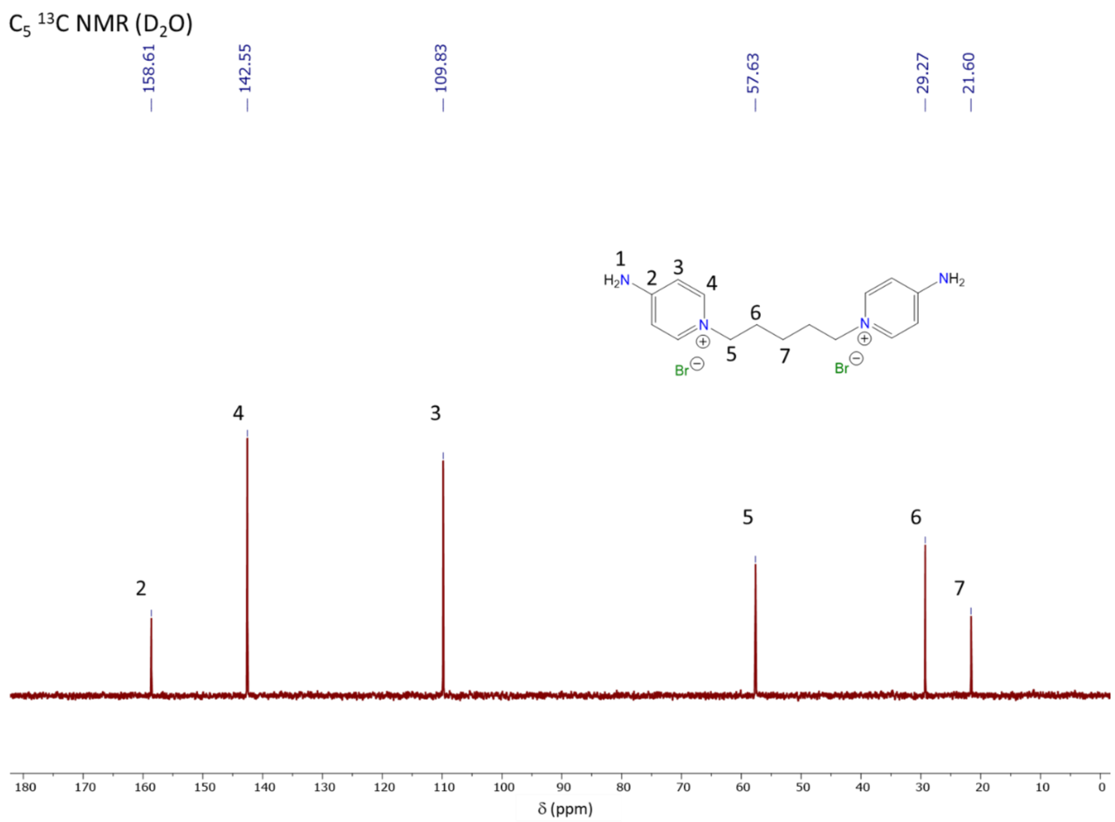

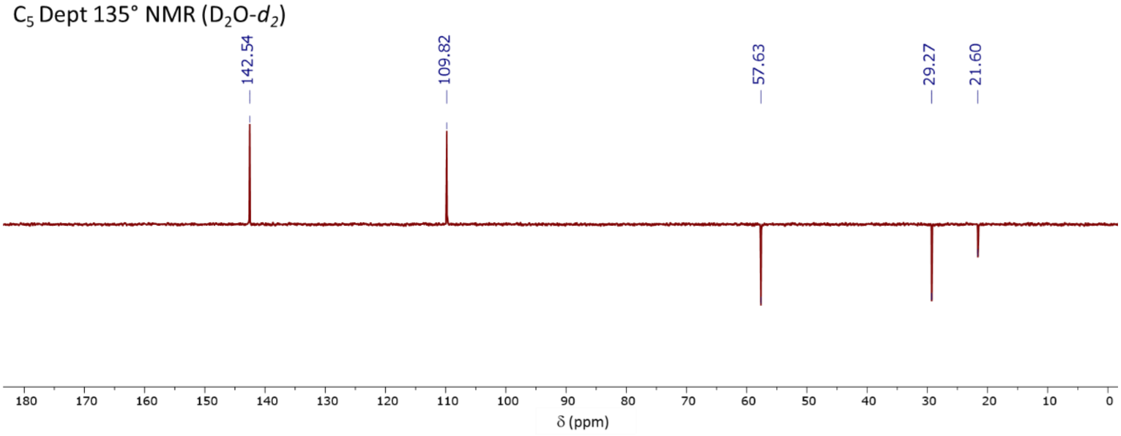

Figure S4. Spectroscopy characterization of 1,1'-(pentane-1,5-diyl)bis(4-aminopyridin-1-ium) bromide (FT-IR, ^{1}H, ^{13}C and Dept 135° NMR spectra).

1,1’-(Hexane-1,6-diyl)bis(4-aminopyridin-1-ium) bromide $[C_6(4\text{-}NH_2Py)_2][Br]_2$ (C_6)

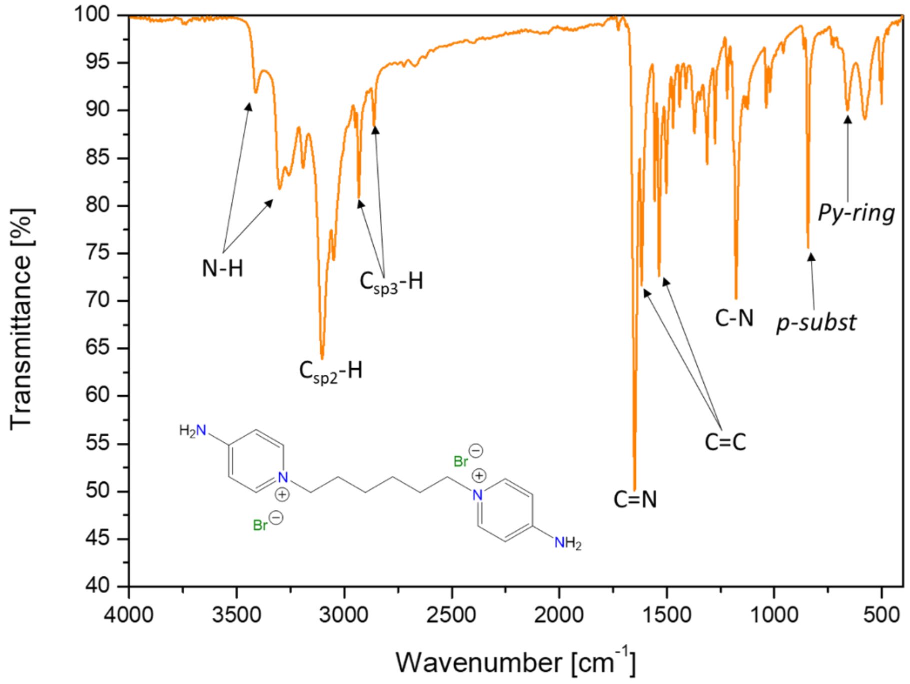

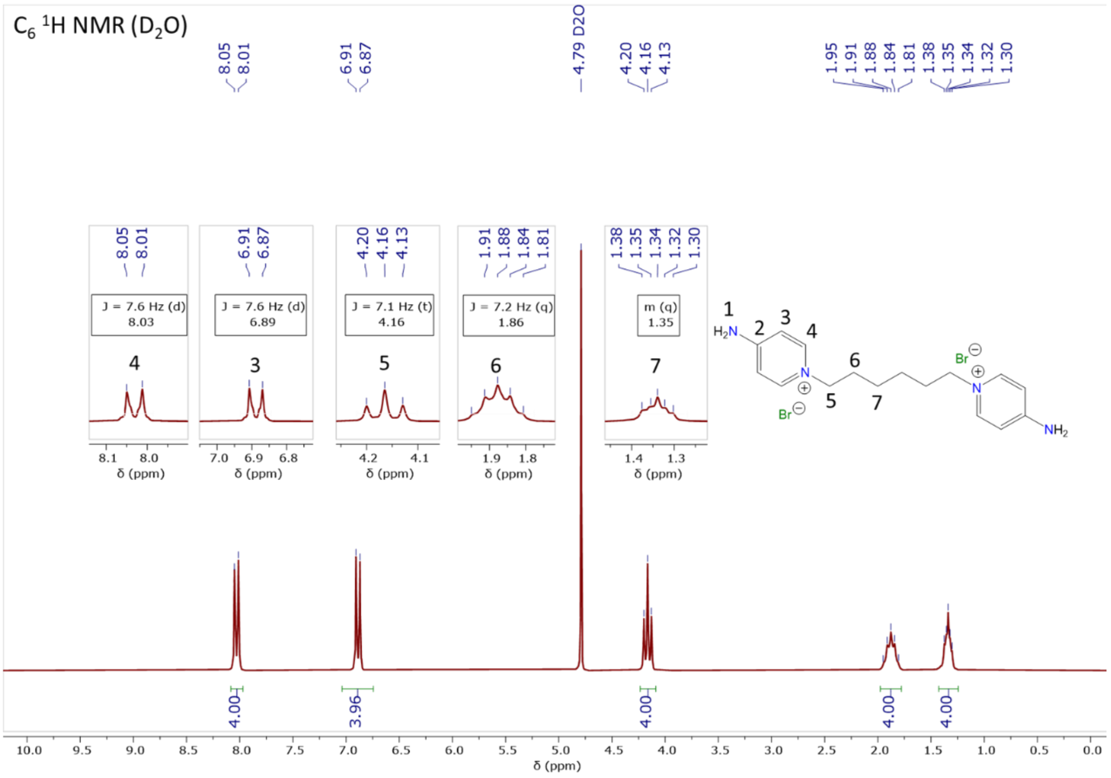

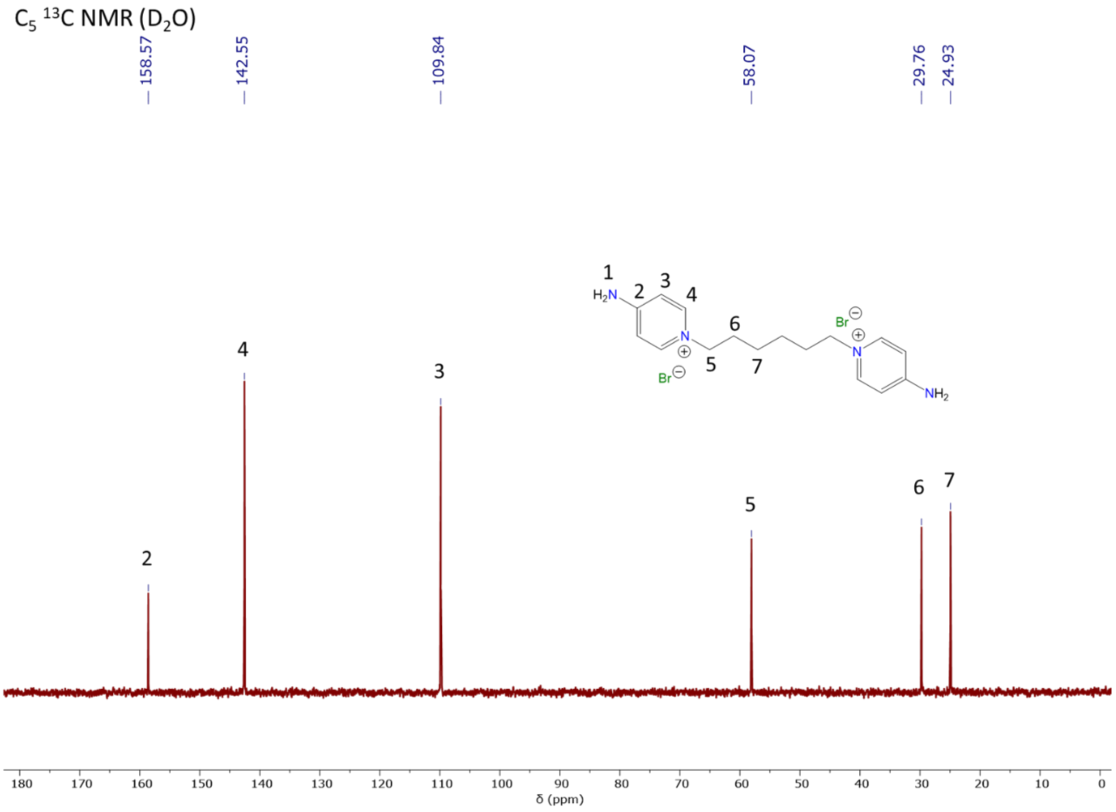

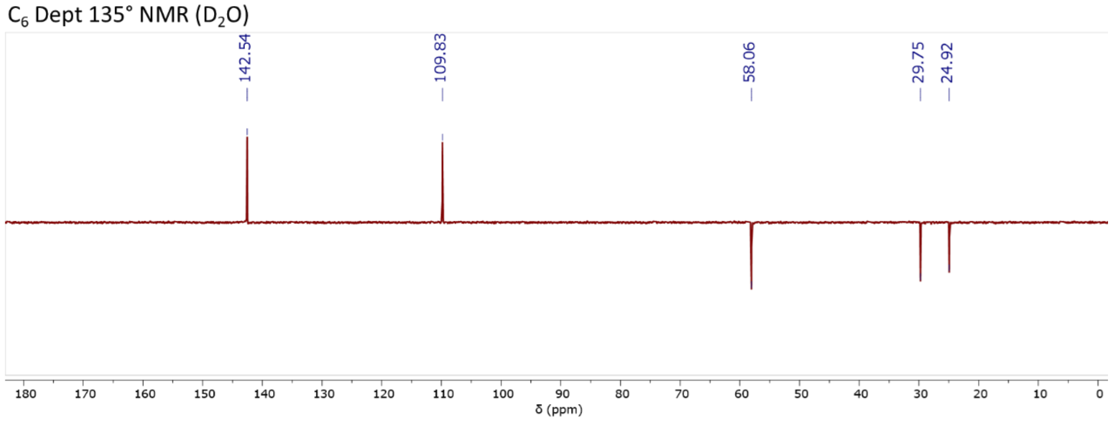

Figure S5. Spectroscopy characterization of 1,1'-(hexane-1,6-diyl)bis(4-aminopyridin-1-ium) bromide (FT-IR, ^{1}H, ^{13}C and Dept 135° NMR spectra).

1,1'-(Octane-1,8-diyl)bis(4-aminopyridin-1-ium) bromide $[C_8(4\text{-}NH_2Py)_2][Br]_2$ (C_8)

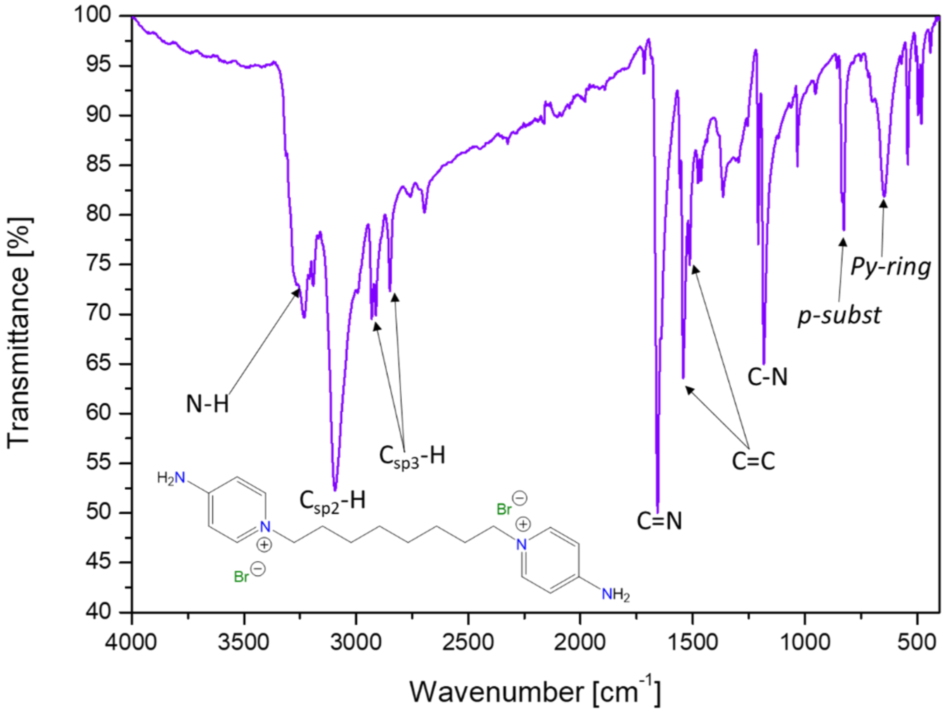

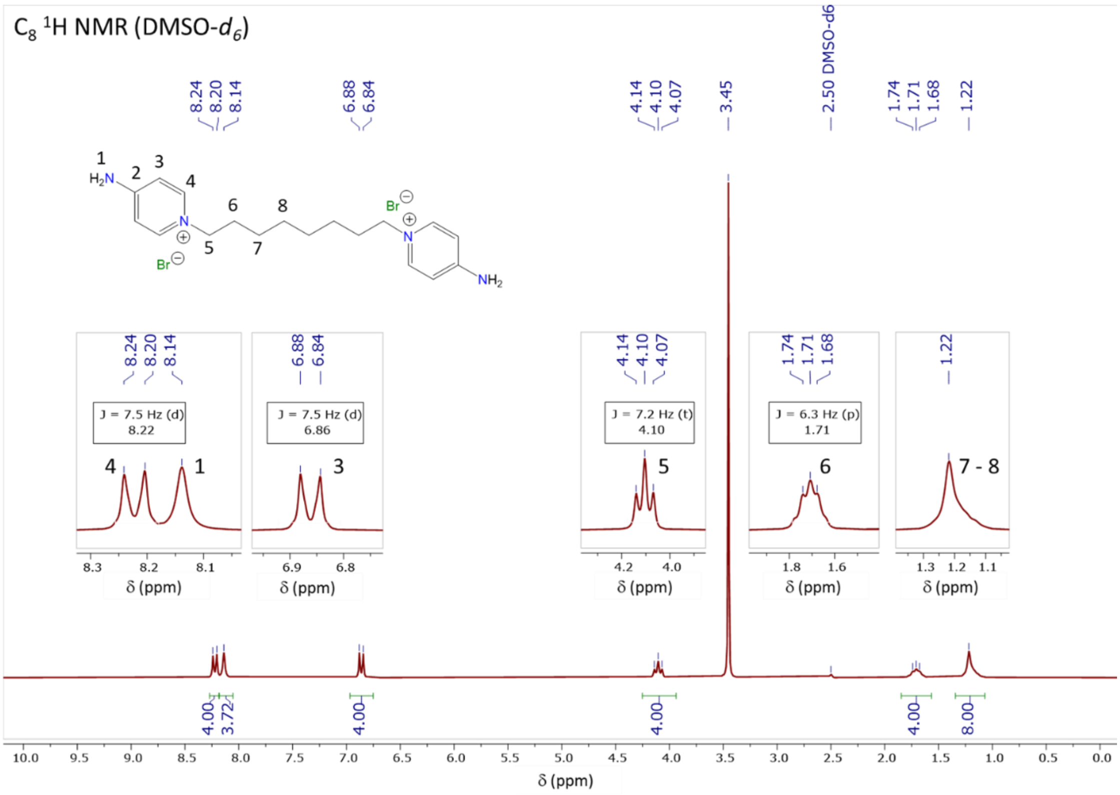

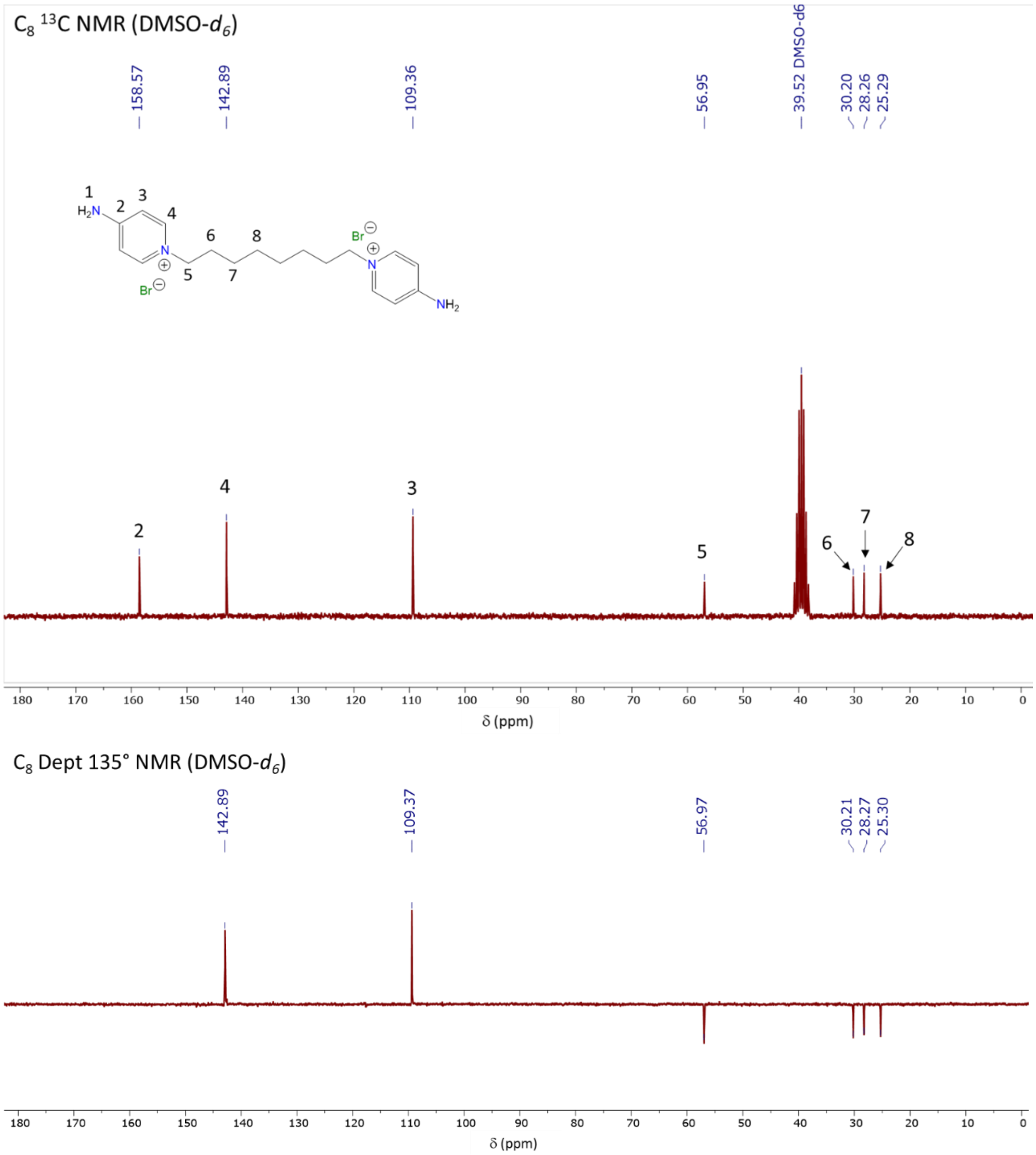

Figure S6. Spectroscopy characterization of 1,1'-(octane-1,8-diyl)bis(4-aminopyridin-1-ium) bromide (FT-IR, ^{1}H, ^{13}C and Dept 135° NMR spectra).

1,1'-(Decane-1,10-diyl)bis(4-aminopyridin-1-ium) bromide [C_{10}(4-NH_2Py)$_2$][Br]$_2$ (C_{10})

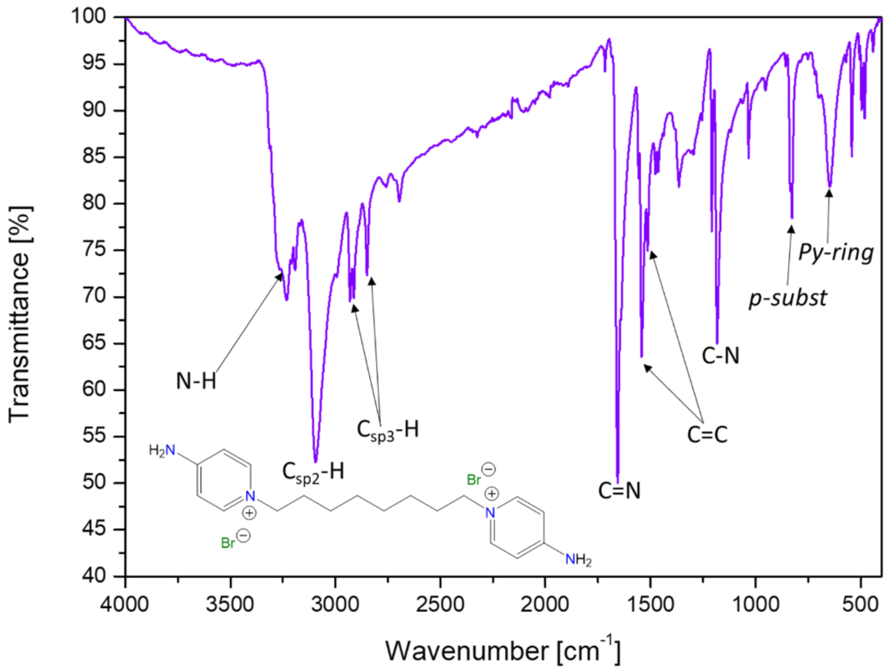

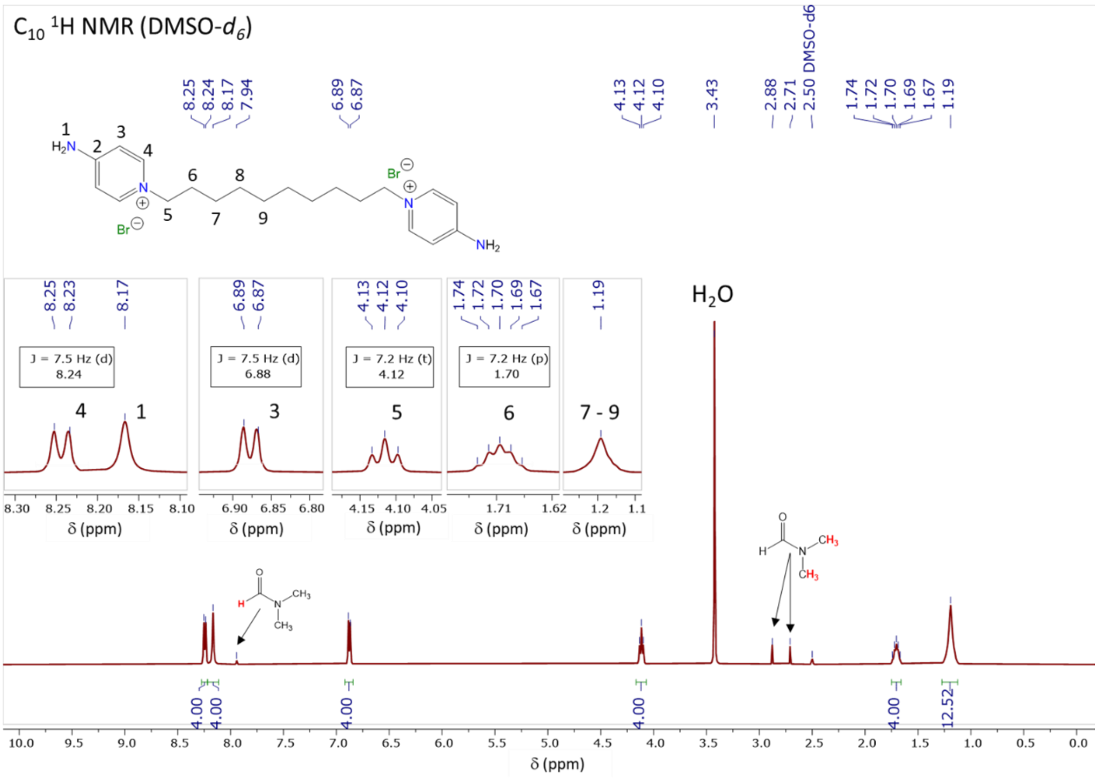

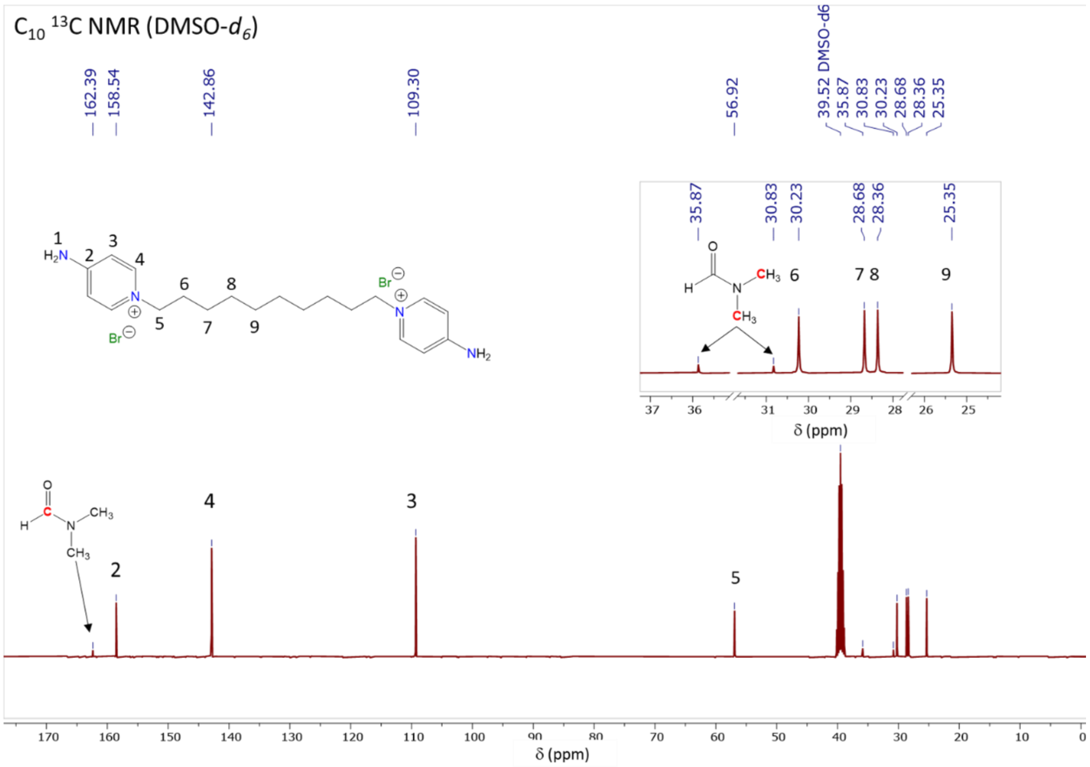

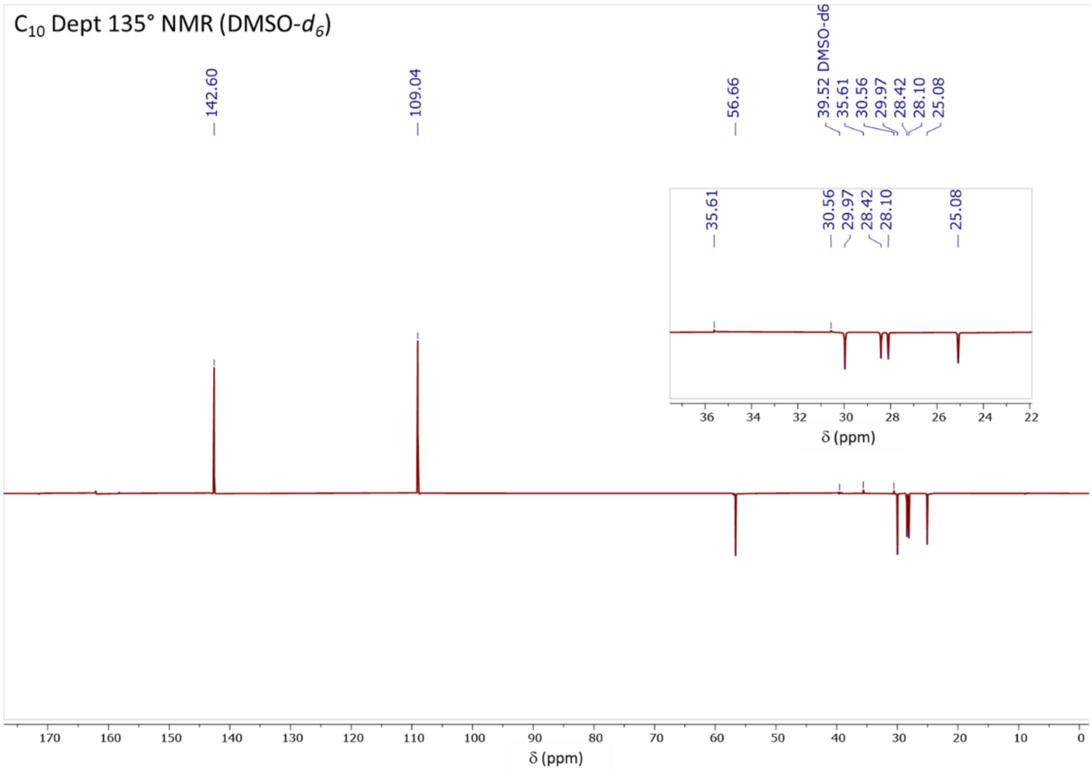

Figure S7. Spectroscopy characterization of 1,1'-(decane-1,10-diyl)bis(4-amino pyridin-1-ium) bromide (FT-IR, ^{1}H, ^{13}C and Dept 135° NMR spectra).

1,1'-(Dodecane-1,12-diyl)bis(4-aminopyridin-1-ium) bromide [$C_{12}(4\text{-}NH_2Py)_2$][$Br$]$_2$ (C_{12})

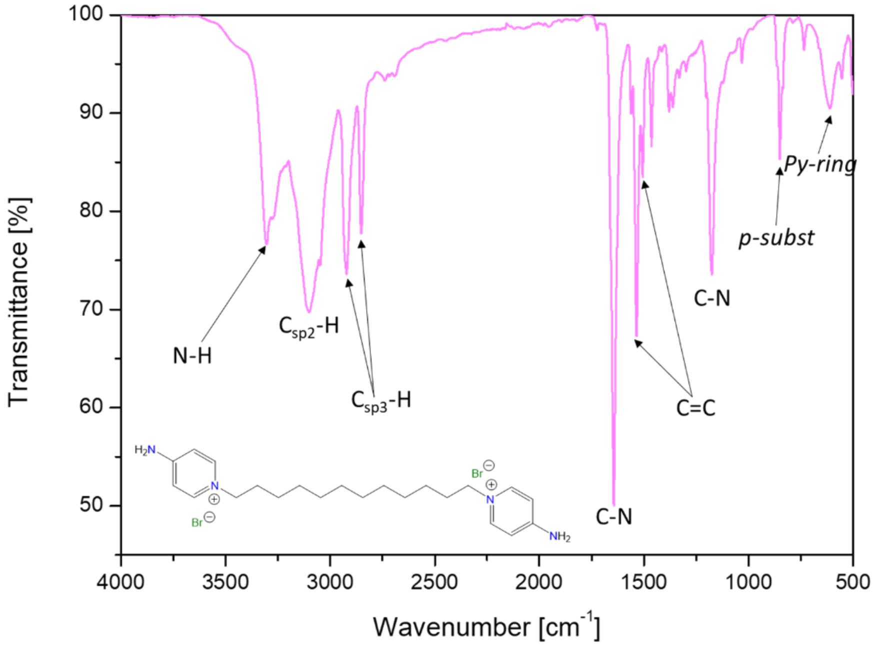

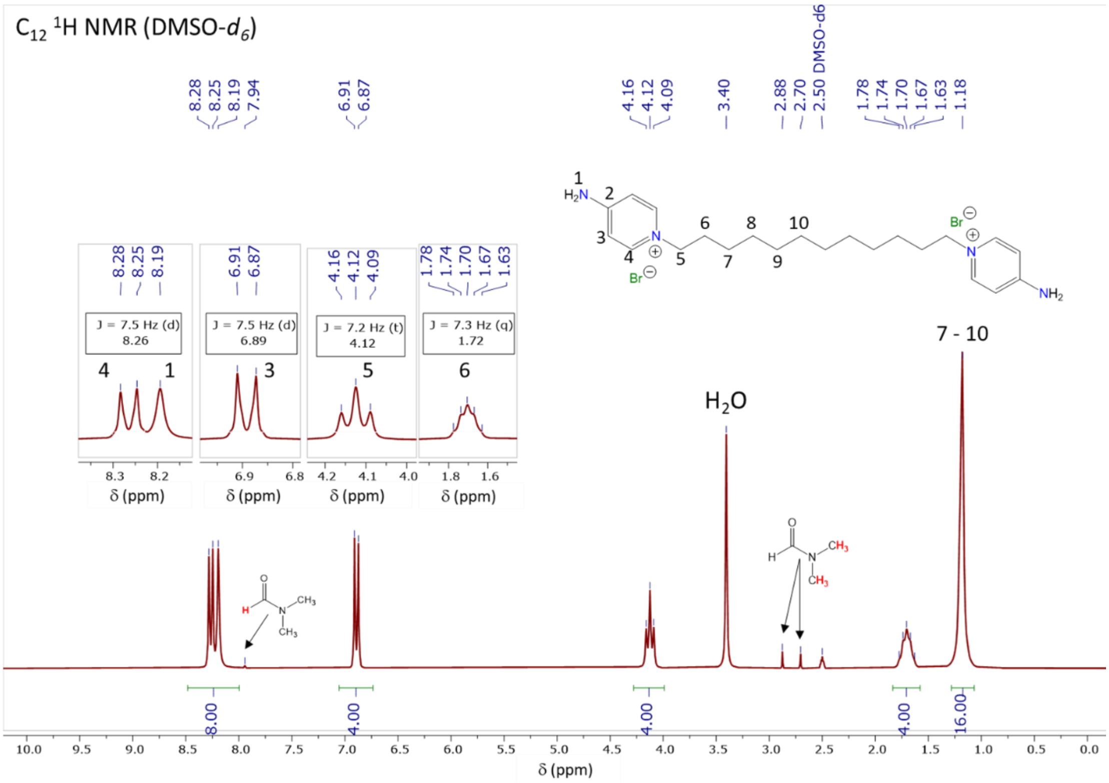

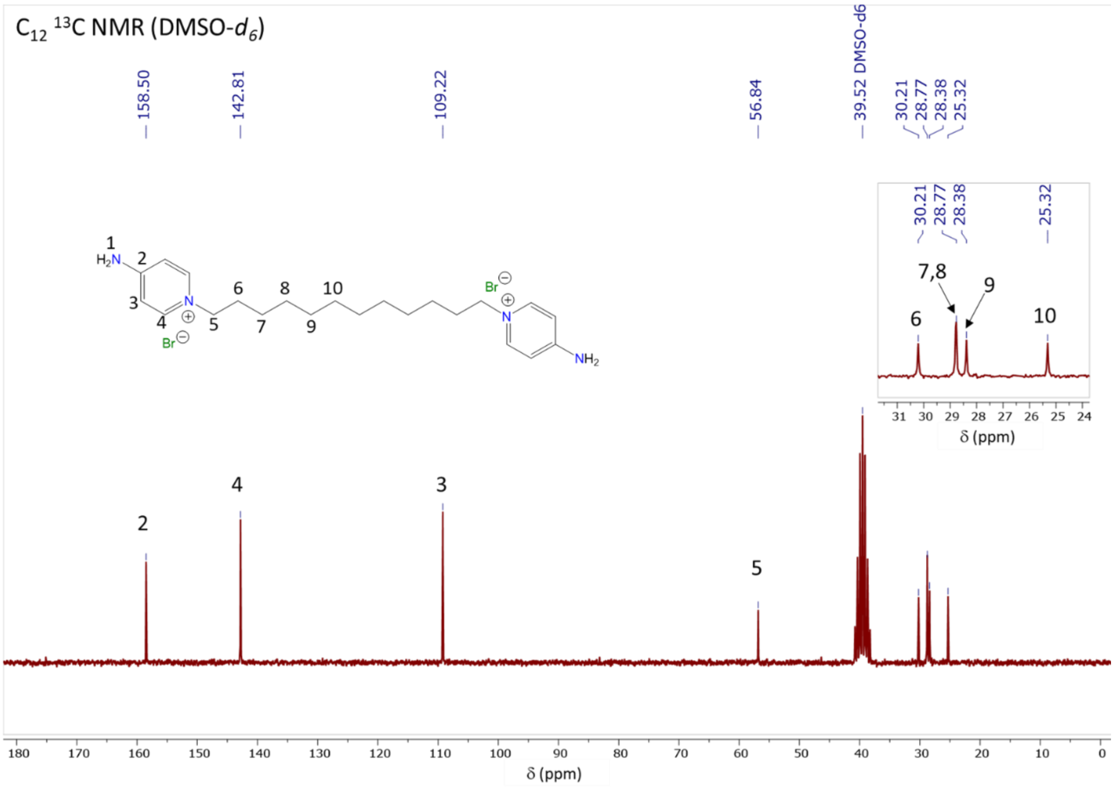

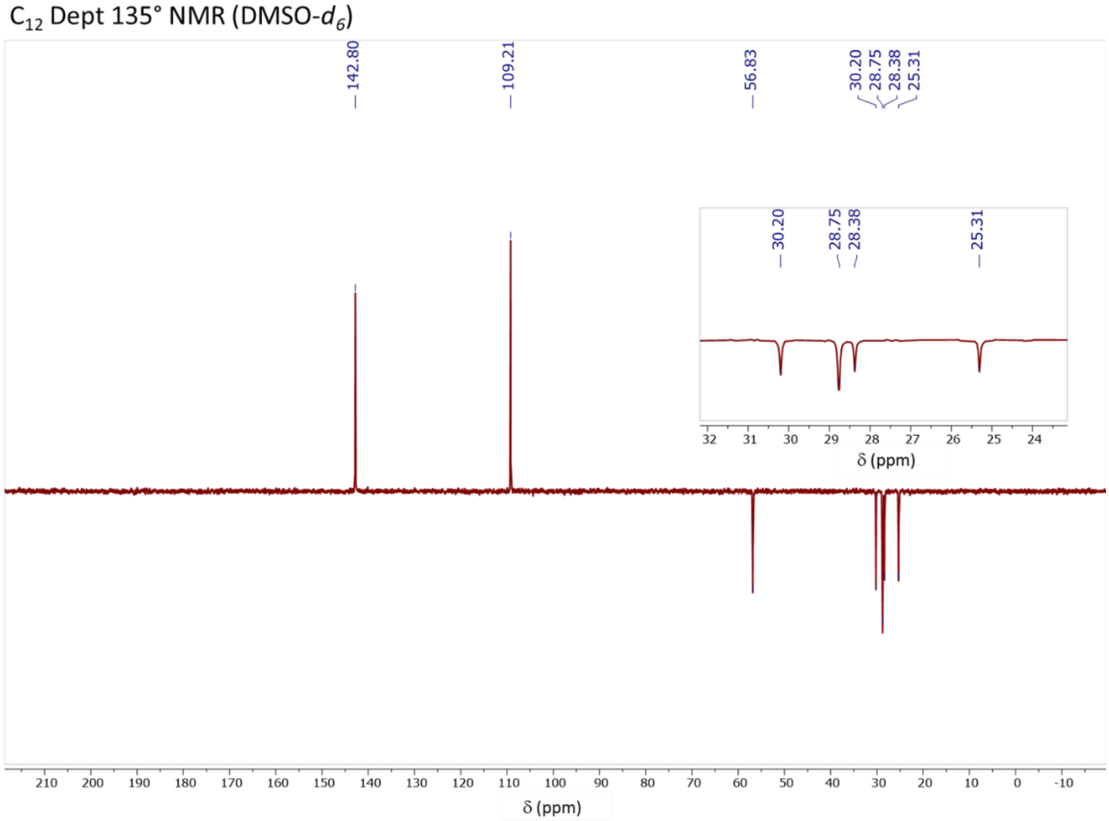

Figure S8. Spectroscopy characterization of 1,1'-(dodecane-1,12-diyl)bis(4-amino pyridin-1-ium) bromide (FT-IR, ^{1}H, ^{13}C and Dept 135° NMR spectra).

1,1'-(1,4-Phenylenebis(methylene))bis(4-aminopyridin-1-ium) bromide $[C_{10}(4\text{-}NH_2 Py)_2][Br]_2$ (C_{Ph})

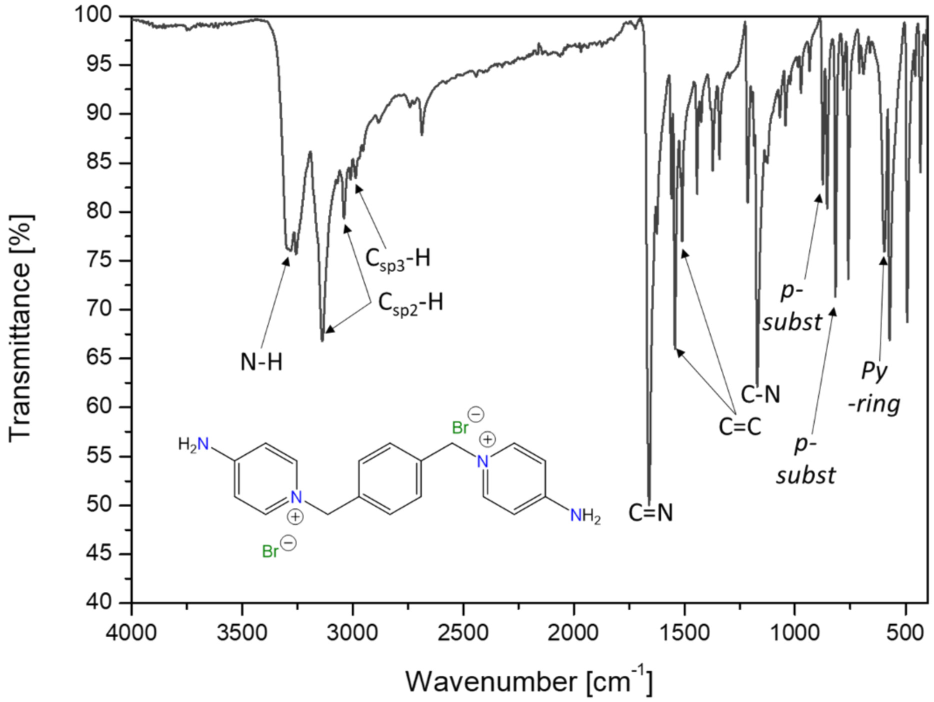

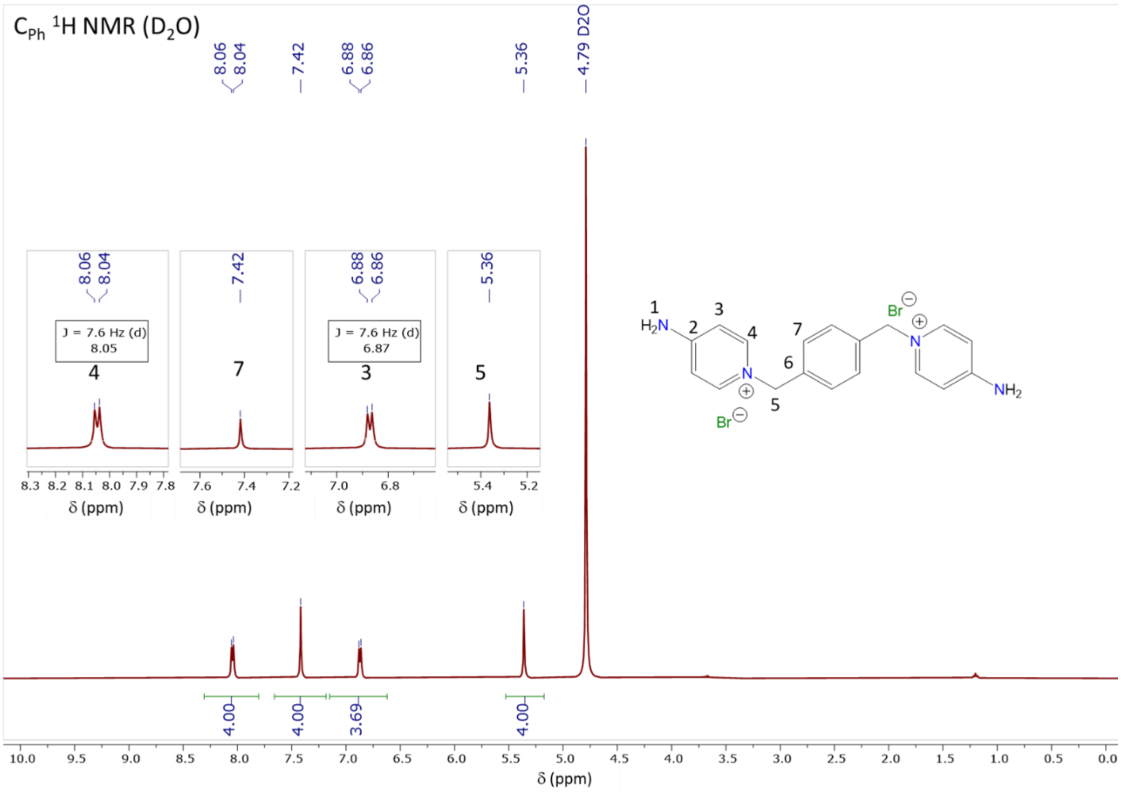

C_{Ph} ^{13}C NMR (D_2O)

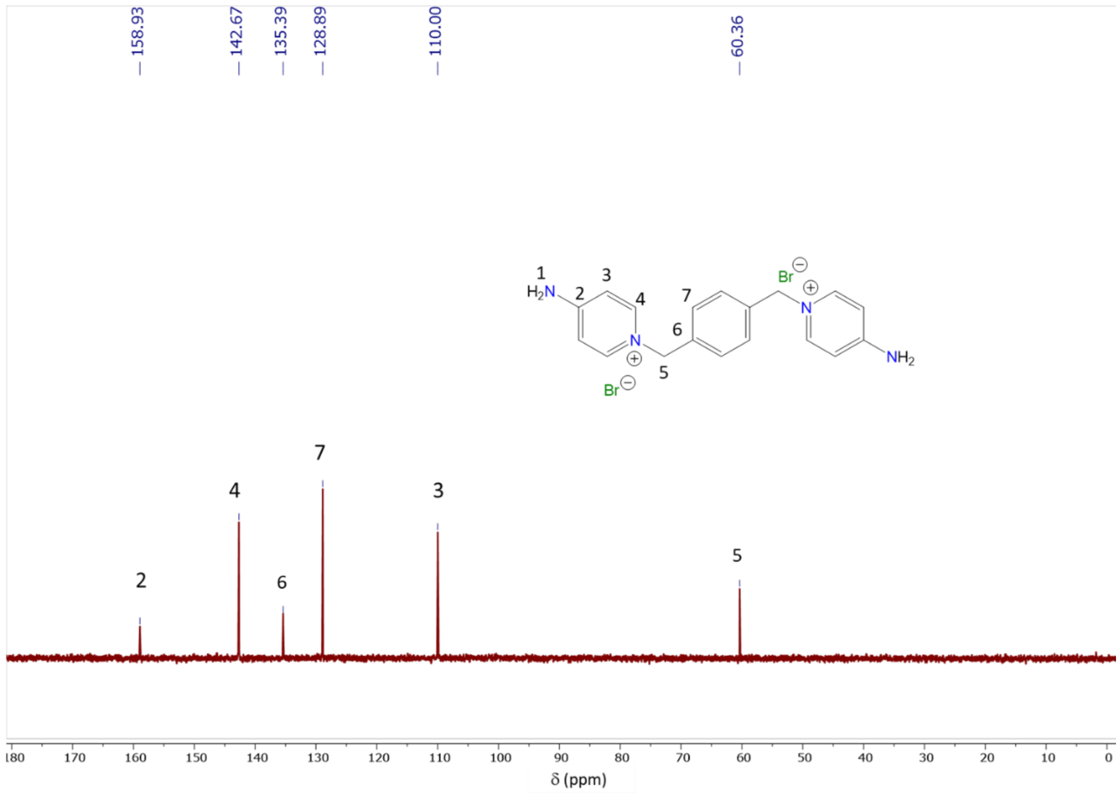

C_{Ph} Dept 135° NMR (D_2O)

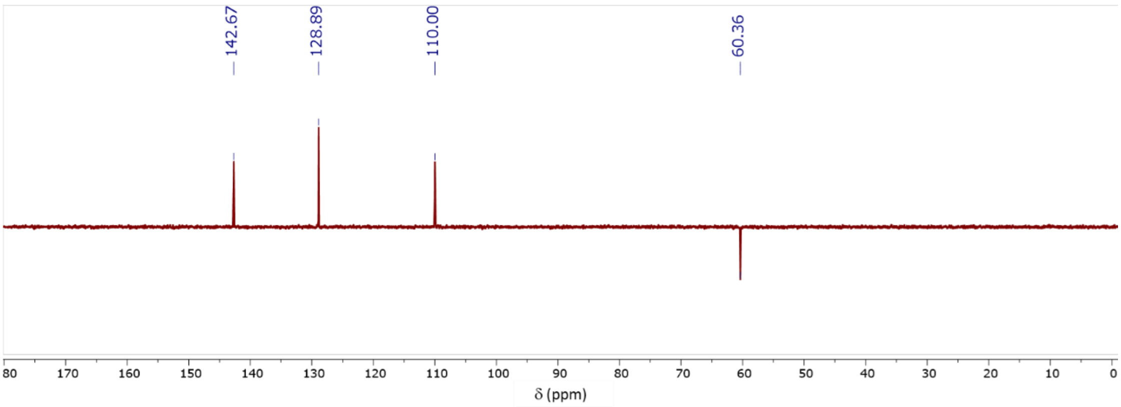

Figure S9. Spectroscopy characterization of 1,1'-(1,4-phenylenebis(methylene))bis(4-aminopyridin-1-ium) (FT-IR, ^1H, ^{13}C and Dept 135° NMR spectra).

Thermal properties

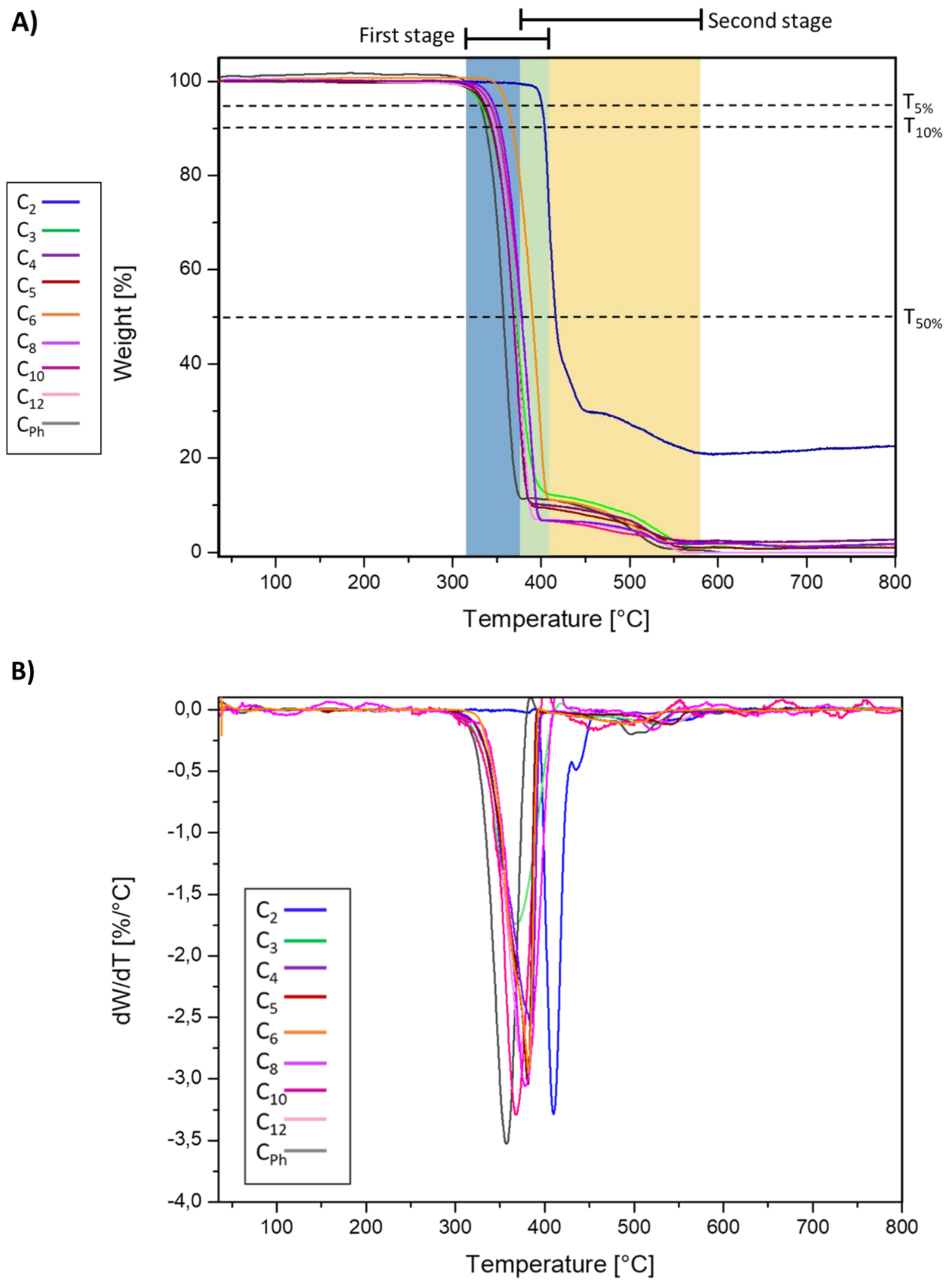

Figure S10. Results of the thermogravimetric analysis conducted on the organic salts. A) TGA curves, and B) DTGA curves.
